# Carbon-Supported PdCu Alloy as Extraordinary Electrocatalysts for Methanol Electrooxidation in Alkaline Direct Methanol Fuel Cells

**DOI:** 10.3390/nano12234210

**Published:** 2022-11-26

**Authors:** Guixian Li, Shoudeng Wang, Hongwei Li, Peng Guo, Yanru Li, Dong Ji, Xinhong Zhao

**Affiliations:** 1 School of Petrochemical Engineering, Lanzhou University of Technology, Lanzhou 730050, China; 2 Basic Research Innovation Group, Project of Gansu Province, Lanzhou 730050, China

**Keywords:** DMFCs, anodic electrocatalysts, palladium-copper alloy, MOR

## Abstract

Palladium (Pd) nanostructures are highly active non-platinum anodic electrocatalysts in alkaline direct methanol fuel cells (DMFCs), and their electrocatalytic performance relies highly on their morphology and composition. This study reports the preparation, characterizations, and electrocatalytic properties of palladium-copper alloys loaded on the carbon support. XC-72 was used as a support, and hydrazine hydrate served as a reducing agent. Pd_x_Cu_y_/XC-72 nanoalloy catalysts were prepared in a one-step chemical reduction process with different ratios of Pd and Cu. A range of analytical techniques was used to characterize the microstructure and electronic properties of the catalysts, including transmission electron microscopy (TEM), X-ray diffractometry (XRD), X-ray photoelectron spectroscopy (XPS), and inductively coupled plasma emission spectroscopy (ICP-OES). Benefiting from excellent electronic structure, Pd_3_Cu_2_/XC-72 achieves higher mass activity enhancement and improves durability for MOR. Considering the simple synthesis, excellent activity, and long-term stability, Pd_x_Cu_y_/XC-72 anodic electrocatalysts will be highly promising in alkaline DMFCs.

## 1. Introduction

With the rapid development of human society, the demand for energy in all countries is increasing, and the accompanying environmental problems are becoming more serious. It has become an imperative development strategy to develop innovative, sustainable, and environmentally friendly energy conversion technologies and devices. Fortunately, Direct Methanol Fuel Cells (DMFCs), as a new energy conversion device with simple structure, high energy density, fast charging, and environmental advantages, can transform direct chemical energy stored in methanol into electricity, which has attracted much attention in recent years [1,2,3,4,5]. At the same time, methanol is a relatively abundant product of the chemical industry, and the development of DMFCs is also considered to be the key to achieving carbon neutrality [6,7,8,9,10]. However, the anodic catalyst with excellent performance and low cost is a challenge for the commercial use of DMFCs. Therefore, it is essential to design and prepare the anode catalyst of DMFCs scientifically [11].

Pt has been verified to be the best catalyst for DMFCs; however, the active site of Pt is easily poisoned by CO_ads_ produced in the process of methanol oxidation, and the methanol oxidation activity on the Pt electrode surface consequently decreases. In order to improve the anti-CO poisoning ability of catalysts, the research of DMFCs anode catalysts mainly focused on Pt and PtM (M = Pd [12], Ru [13], Fe [14], Co [15], Ni [16], Cu [17], Sn [18], and Ag [19]). Some studies have shown that there are two mechanisms of bifunctional mechanism and electronic effect in improving the anti-CO poisoning ability of Pt-based alloy catalysts. The bifunctional mechanism holds that methanol takes the adsorption–dehydrogenation processes on the surface of Pt. The second metal or metal oxide which is introduced promotes the dissociation of the electrolyte to form more OH_ads_, which is conducive to faster oxidation removal of CO_ads_ adsorbed on Pt [20,21]. On the other hand, the second metal reduces the adsorption energy of CO_ads_ on the Pt surface by affecting the electronic properties of Pt, thus inhibiting the adsorption of CO_ads_ on the Pt surface. The Pd-based catalyst is more abundant, has higher electrocatalytic activity for small alcohol molecules in alkaline media, and is resistant to CO poisoning. Thus, it can be used as a substitute for Pt-based anode catalysts [22].

Therefore, in order to further improve the electrocatalytic activity and economical utilization of the Pd catalyst, the second metal is usually introduced to form the alloy PdM (M = Cu [23], Co [24], Ag [25], Fe [26], and Sn [27]). Comparing it with a single metal, the bimetallic catalyst exposed to more active sites had higher activity and stability, owing to the shift of the d-band center of Pd, which is caused by the electronic effects and geometric changes of the catalyst. In addition, the support with a large specific surface area can provide a channel for electron transfer, such as carbon black, carbon nanotubes, and graphene, thus maintaining the excellent conductivity and performance of the catalyst.

In recent years, palladium copper alloy nanoparticles have attracted much attention due to their excellent small molecule oxidation properties of alcohols and ability to resist CO_ads_ poisoning. The reason for this is that the d-band center of Pd shifts after the alloy formation between Cu and Pd, and the addition of copper reduces the binding energy of Pd^n+^. In contrast, Pd increases the binding energy of Cu^n+^, which weakens the adsorption of CO_ads_ on the catalyst surface.

Recently, Ye et al. [28] reported a PdCu catalyst with an unique core–shell structure. In an alkaline medium, the catalyst has excellent catalytic performance for methanol oxidation reaction (MOR), and the enhanced activity is rigorously ascribed to the adsorption strength of OH_ads_, which is good for the removal of adsorbed COads and increases the ability of CO_ads_ to resist CO_ads_ poisoning. Shih et al. [23] studied porous PdCu NPs for electrocatalytic oxidation of methanol with excellent electrochemical activity, greater stability, and lower cost-effectiveness. The copper content of a catalyst is known to have a significant effect on its morphology and catalytic activity. Additionally, the copper content controls the morphology and affects the catalytic activity toward the MOR in alkaline media. This is an important discovery, because it can help to optimize the performance of catalysts for various applications. Saleem et al. [29] prepared an element segregation phenomenon in two-dimensional (2D) core–shell nanoplates, which showed superior electrocatalytic activity and stability towards the MOR compared to the commercial Pt/C catalyst. Unfortunately, the complex synthesis process impedes its practical application in DMFCs. Therefore, it is urgent to design and synthesize an efficient MOR catalyst using convenient, rapid, and eco-friendly synthetic methods.

Based on the above views, in this work, we synthesized PdCu nanoalloys on Vulcan XC-72 support by means of the liquid reduction method, which can synthesize many alloy catalysts in a short time under mild conditions, and meets the requirements of green chemistry. In addition, we synthesized a series of PdCu nanoalloys with different atomic ratios to study the influences of Cu addition on the electronic environment and the lattice strain of the catalysts.

## 2. Materials and Methods

### 2.1. Materials and Reagents

Palladium chloride PdCl_2_ (>99.9%); ethylene glycol (>99.8%), hydrazine hydrate (>85%), XC-72, concentrated hydrochloric acid (36~38%), copper nitrate solution (>99%), and all other reagents were of analytical grade and purchased from MACKLIN (Shanghai, China) without further purification.

### 2.2. Preparation of Catalysts

The supported Pd_x_Cu_y_/XC-72 (x + y = 5) nanoalloy catalysts with different compositions were prepared by one-step chemical reduction, with hydrazine hydrate as a reducing agent and controlling the total metal loading at 30%. Typically, taking Pd_1_Cu_1_/XC-72 as an example, 50.0 mg XC-72 was accurately weighed and dispersed in 200 mL ethylene glycol for 30 min by ultrasound, and 2 mL hydrazine hydrate was added to the mixture under stirring. Then, 11.41 mL of H_2_ PdCl_4_ (1 mg/mL) and 8.011 mg Cu(NO_3_)_2_·3H_2_O mixed precursor solution was added to it by dropping, and the mixture was reduced by stirring for two hours, followed by filtration, washing, and drying at 70 °C overnight to obtain Pd_1_Cu_1_/XC-72.

### 2.3. Physical Property Characterization

In order to conduct an X-ray diffraction (XRD, Bruker D8 Advance, Germany) analysis, a Bruker D8 Advance, equipped with Cu target Kα radiation as the X-ray source (λ = 0.15404 nm), was employed. Transmission electron microscopy (TEM, FEI Talos F200x, USA) and high-resolution transmission electron microscopy (HRTEM, FEI Talos F200x, USA) were also performed on a JEOL 2100F TEM with an accelerating voltage of 200 kV. Inductively coupled plasma-atomic emission spectroscopy (ICP-OES, Agilent 5110(OES), USA) was a powerful analytical tool that could measure a wide range of elements in a sample. In this study, ICP-OES was used to measure the concentration of elements in the sample. This information was then used to understand the chemical composition of the sample. To this end, a certain amount of the sample was digested by microwave with aqua regia at 200 °C for 1 h. This process was repeated five times until it was clear and transparent, to ensure that the solids were completely digested. Then, the as-obtained solution was volume-constant at 25 mL and diluted 100 times. Moreover, in order to obtain the electronic structure information of the catalyst surface, the Pd_x_Cu_y_/XC-72 was analyzed by X-ray photoelectron spectroscopy (XPS, Thermo Scientific K-Alpha, America). The as-synthesized materials were evaluated for electrochemical characterization using cyclic voltammetry (CV) and chronoamperometry (CA).

### 2.4. Electrochemical and Physical Characterization

The electrochemical performance of electrocatalysts were evaluated by workstation (CHI 660E) of a three-electrode system. The platinum electrode was the counter electrode, the calomel saturated electrode (SEC) was the reference electrode, and the glassy carbon electrode (GCE, 4.0 mm in diameter, 0.1256 cm [2]) coated with catalyst was the working electrode. The working electrode was prepared as follows: 2.0 mg of catalyst was accurately weighed, and 450 μL absolute ethanol, 50 μL 5% Nafion membrane solution, and 500 μL ultrapure water were successively added to the catalyst. It was then dispersed by ultrasound for 0.5 h to form a uniform inked mixture. Next, 5 μL was removed and dropped onto the surface of the glassy carbon electrode, which was allowed to dry naturally before testing. Cyclic voltammetry (CV) was used to test the activity of electrocatalytic methanol oxidation and the electrochemical active area (ECSA) in an alkaline environment. The MOR activity test electrolyte was 1 M KOH + 1.0 M CH_3_OH solution saturated with N_2_, and the ECSA test was placed into 1 M KOH solution saturated with N_2_. The scanning speed was 50 mV/s, and the potential range was −0.9–0.3 V (vs. SCE). In this work, a chronoamperometry experiment was used to evaluate the stability of the catalyst. The specific operating conditions were as follows: a chronoamperometry test was performed at −0.2 V constant potential, and rapid scanning was performed at 0.1 V/s scanning speed. All above tests were performed at room temperature.

## 3. Results

### 3.1. Physical Characterization of Pd_x_Cu_y_/XC-72

**Figure 1:**
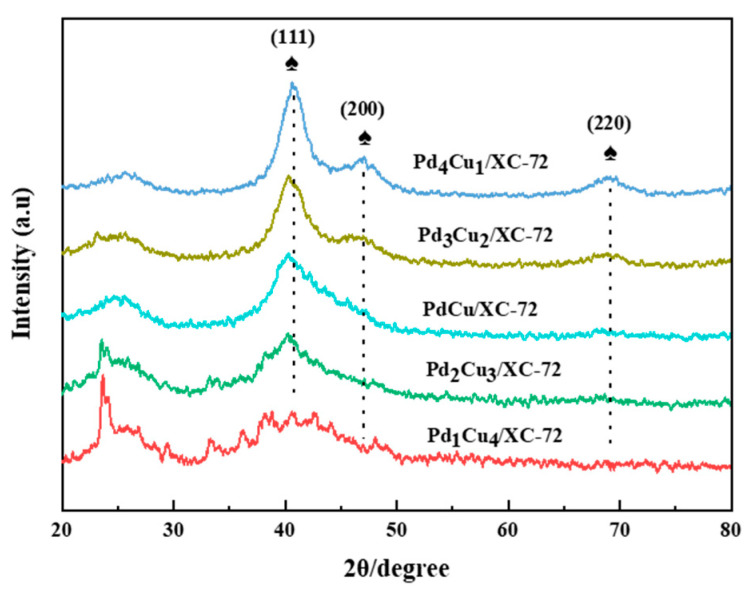
XRD pattern of the electrocatalysts.

Figure 1 indicates the XRD profile of the as-synthesized Pd_x_Cu_y_/XC-72. The XRD profile reveals that the PdCu NPs are well crystallized, as observed from the three prominent diffraction peaks. The three main diffraction peaks appear at 2θ = 40.6°, 46.7°, and 68.2° index to the (111), (200), and (220) crystal planes of face-centered cubic (fcc) Pd NPs. The Pd (111) shift peak is shown in Figure 1. After Cu incorporation, the diffraction peak at 40.6°, corresponding to the Pd (111) plane, slightly shifted to the higher values (namely, 40.61°, 40.26°, 40.28°, 40.20°, 40.00°). This shift may be due to the Pd lattice contraction, owing to the smaller radius of Cu (0.128 nm) compared to that of Pd (0.137 nm), indicating the formation of successful PdCu alloy NPs [30].

**Figure 2:**
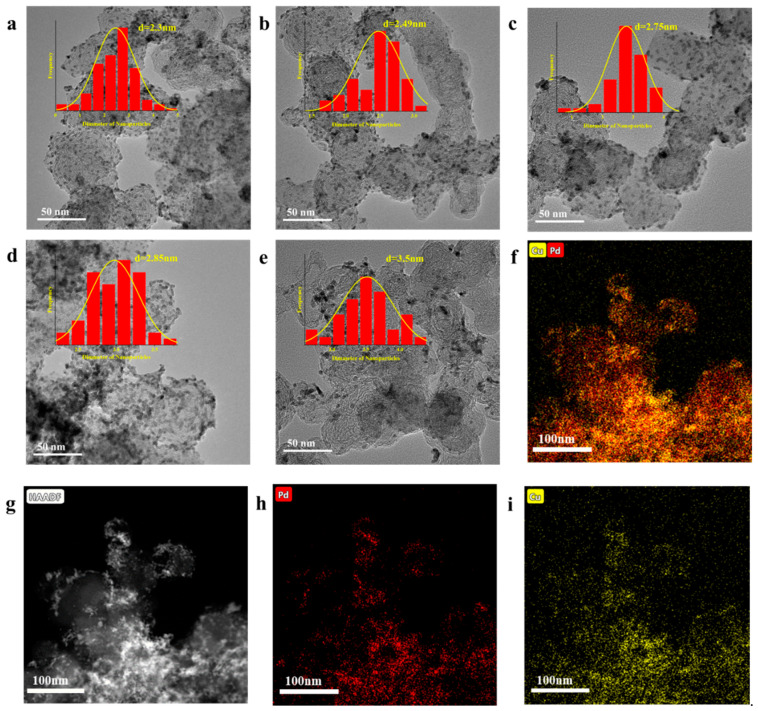
TEM Micrographs of Pd_x_Cu_y_/XC-72 (**a**) Pd_1_Cu_4_/XC-72 (2.3 ± 1 nm), (**b**) Pd_2_Cu_3_/XC-72 (2.49 ± 1 nm), (**c**) PdCu/XC-72 (2.75 ± 1 nm), (**d**) Pd_3_Cu_2_/XC-72 (2.85 ± 2 nm), and (**e**) Pd_4_Cu_1_/XC-72 (3.5 ± 1 nm). (**f**–**i**) EDX element maps of Pd_3_Cu_2_/XC-72.

The dispersion of several catalysts with different PdCu ratios is reflected in the TEM images of Pd_x_Cu_y_/XC-72 in Figure 2. It can be seen that the catalysts formed with our synthesized are well-dispersed, with a uniform size distribution, and the particle size of the formed catalysts gradually rises with the increase in the Pd ratio.

**Figure 3:**
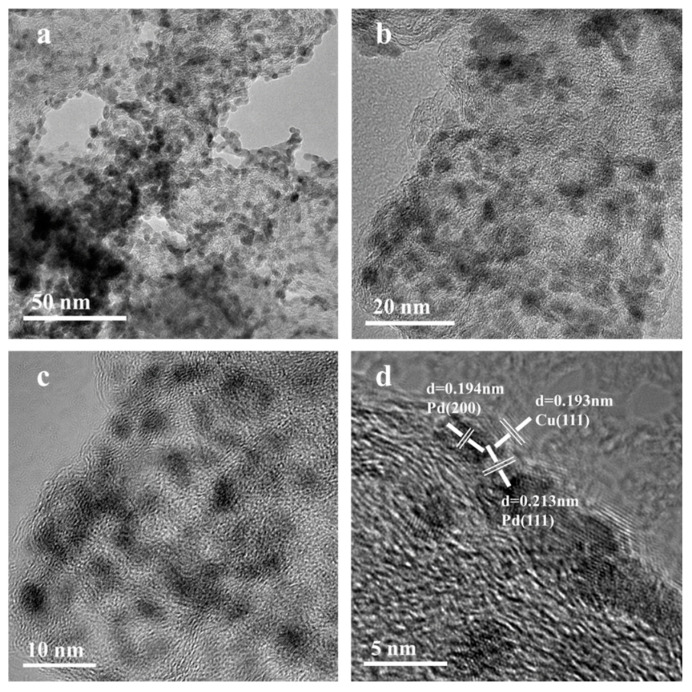
HRTEM image of Pd_3_Cu_2_/XC-72 (**a**) 50 nm, (**b**) 20 nm, (**c**) 10 nm, (**d**) 5 nm.

Figure 3 shows the HRTEM profile of the Pd_3_Cu_2_/XC-72. It can be seen that the metal is dispersed more evenly on the carbon support, and in the Figure 3c, the lattice fringes of the Pd(111) plane, Pd (200) plane, and Cu (111) plane can be clearly seen, which further verifies the formation of its alloy [31].

Moreover, we used the microwave digestion technique combined with inductively coupled plasma optical emission spectrometry (ICP-OES) analysis. Table 1 shows that the Pd metal content of Pd_1_Cu_4_/XC-72, Pd_2_Cu_3_/XC-72, PdCu/XC-72, Pd_3_Cu_2_/XC-72, and Pd_4_Cu_1_/XC-72 is 7.6, 12.4, 18.2, 19.7, and 25.8 wt%, respectively. In addition, the Cu metal content of Pd_1_Cu_4_/XC-72, Pd_2_Cu_3_/XC-72, PdCu/XC-72, Pd_3_Cu_2_/XC-72, and Pd_4_Cu_1_/XC-72 is 15.4, 11.1, 10.2, 6.9, and 3.5 wt%, respectively. This generally agrees with the ratio of PdCu alloy in our experimental design.

**Figure 4:**
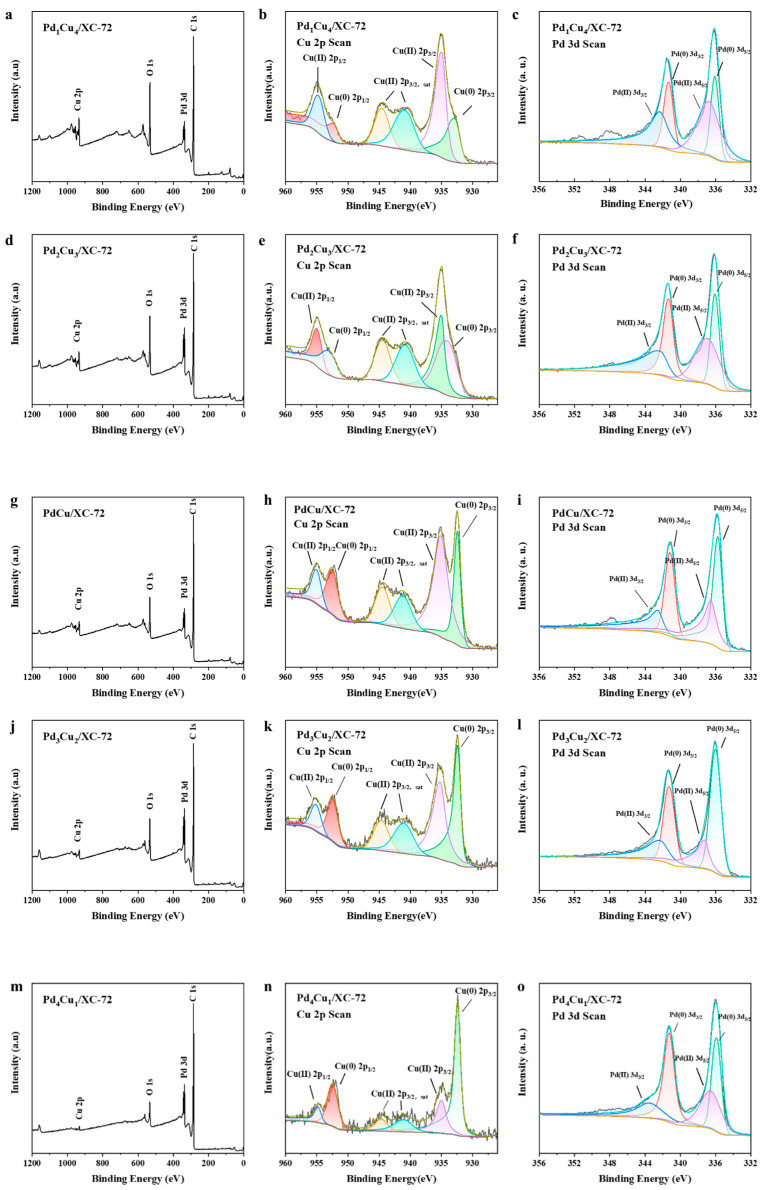
XPS data showing the wide spectra of (**a**–**c**) Pd_1_Cu_4_/XC-72 wide spectra, Cu 2p, and Pd 3d; (**d**–**f**) Pd_2_Cu_3_/XC-72 wide spectra, Cu 2p, and Pd 3d; (**g**–**i**) PdCu/XC-72 wide spectra, Cu 2p, and Pd 3d; (**j**–**l**) Pd_3_Cu_2_/XC-72 wide spectra, Cu 2p, and Pd 3d; (**m**–**o**) Pd_4_Cu_1_/XC-72 wide spectra, Cu 2p, and Pd 3d.

The composites were further examined by XPS in order to learn more about the surface electronic state of Pd_x_Cu_y_/XC-72 (Figure 4). To ascertain the valence state of Pd in the hybrids, the XPS spectra of Pd 3d were also gathered. Metallic Pd^0^ is responsible for the Pd 3d5/2 peak at 335.2 and the 3d3/2 peak at 340.5 eV, and also the Pd 3d5/2 peak at 342.4 eV and 3d5/2 peak at 337.2 eV assigned to Pd^2+^. It can be seen that the majority of the metal precursors are successfully reduced and loaded onto the support by comparing the quantities of Pd^0^ and Pd^2+^. According to the Cu 2p spectra, the Pd_x_Cu_y_/XC-72 peaks at 952.56 and 932.7 eV, respectively, correspond to Cu^0^ 2p1/2 and 2p3/2; the peaks at 953.7 and 933.6 eV, respectively, correspond to Cu^2+^ 2p1/2 and 2p3/2. The peaks at 944.8 and 941.9 eV, correspond to Cu^2+^ 2p3/2 satellite peaks. The overall intensity of the Pd peaks decreases with the addition of Cu, indicating less Pd exposure in the bimetallic.

**Figure 5:**
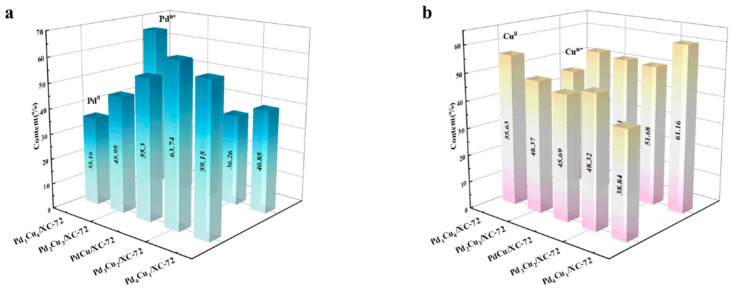
(**a**) The corresponding Pd^0^ and Pd^n+^ contents of Pd_1_Cu_4_/XC-72, Pd_2_Cu_3_/XC-72, PdCu/XC-72, Pd_3_Cu_2_/XC-72, and Pd_4_Cu_1_/XC-72, as well as (**b**) the corresponding Cu^0^ and Cu^n+^ contents of Pd_1_Cu_4_/XC-72, Pd_2_Cu_3_/XC-72, PdCu/XC-72, Pd_3_Cu_2_/XC-72, and Pd_4_Cu_1_/XC-72.

Area ratios for various Pd_x_Cu_y_/XC-72 catalysts are shown in Figure 5. Since Pd^n+^ to Pd^0^ and Cu^n+^ to Cu^0^ are the alloys formed during reduction, other metals will change to create a relatively stable state during the alloy formation process. At this point, low-valence Cu 2p electrons will be transferred to Pd, reducing the positive state of Pd^n+^ while increasing the valence state of low-valence Cu, resulting in a gradual rise in the concentration of Cu^n+^.

However, Pd_3_Cu_2_/XC-72 has the highest value of Pd^0^ and Cu^n+^ content. There is also a change in the binding energy of a fraction of the Pd, represented by the broadening of the peaks. The electronic structure of the surface Pd atoms is modified by alloying with the Cu atoms as the Cu content increases and the binding energy shifts to a higher value. These results further confirm that the alloy of Pd_x_Cu_y_ nanoparticles supported on XC-72 has been successfully synthesized by the chemical reduction method. In addition, the presence of Cu^2+^ may be caused by the surface oxidation or chemisorption of environmental oxygen during the synthesis process, as indicated by the shake-up satellite (sat.) peaks at the high binding energy side of the Cu 2p3/2 and Cu 2p1/2. The XPS spectra of the other catalysts are shown in Figure 4. The characteristic peaks for each catalyst are the same as the Pd_x_Cu_y_/XC-72 XPS results.

### 3.2. Electrochemical Tests for MOR (Three-Electrode Cell)

**Figure 6:**
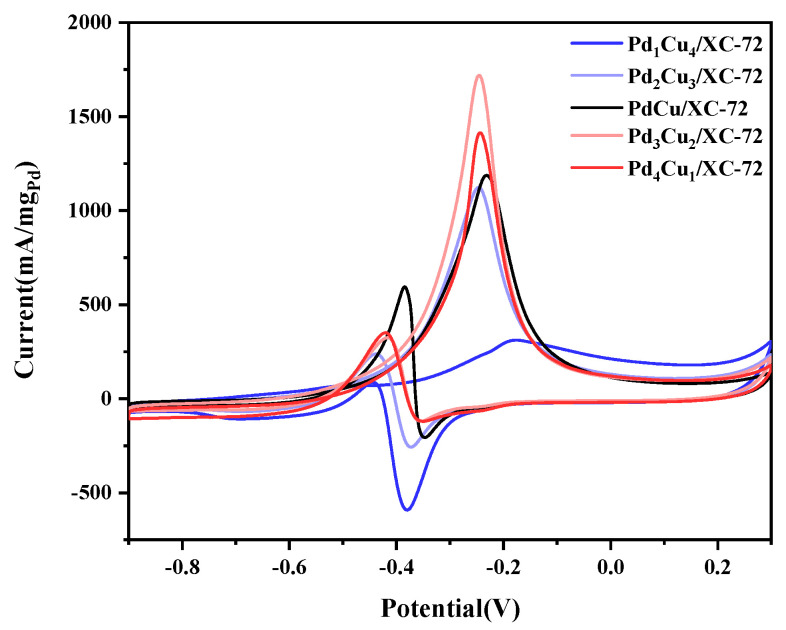
CVs in 1.0 M KOH + 1.0 M CH_3_OH-purged N_2_ at 50 mVs^−1^.

As is summarized in Figure 6, the electrochemical performance of the five electrocatalysts is shown. Cyclic voltammetry (CV) was performed on each of the Pd_1_Cu_4_/XC-72, Pd_2_Cu_3_/XC-72, PdCu/XC-72, Pd_3_Cu_2_/XC-72, and Pd_4_Cu_1_/XC-72 electrodes in N_2_-saturated 1.0 M KOH and 1.0 M CH_3_OH. It can be seen that there are obvious differences in electrocatalytic activities with different amounts of Cu. The results depict that Pd_3_Cu_2_/XC-72, the maximum value, was obtained (1719 mA·mg^−1^ Pd). Therefore, combined with XPS (Figure 4) and XRD (Figure 1) data, we can conclude that Pd_3_Cu_2_/XC-72 exhibits excellent catalytic performance due to its excellent electronic structure. In addition, it was found that the performance of the catalyst synthesized in this work was much higher than that of the catalysts synthesized in other works (Table 2).

Generally, the ratio between the peak current in the forward (*I_f_*) and the backward scan (*I_b_*) is the measure for the tolerance of the catalyst against CO poisoning, with a higher *I_f_:I_b_* indicating higher tolerance. In our experiment, Pd_3_Cu_2_/XC-72 displays a higher *I_f_:I_b_*, which reflects its higher CO tolerance (Table 3). In other words, *I_f_:I_b_* is positively correlated with the area ratio of Pd^0^ and Cu^n+^, which proves that higher Pd^0^ and Cu^n+^ in the catalyst results in higher anti-CO performance.

**Figure 7:**
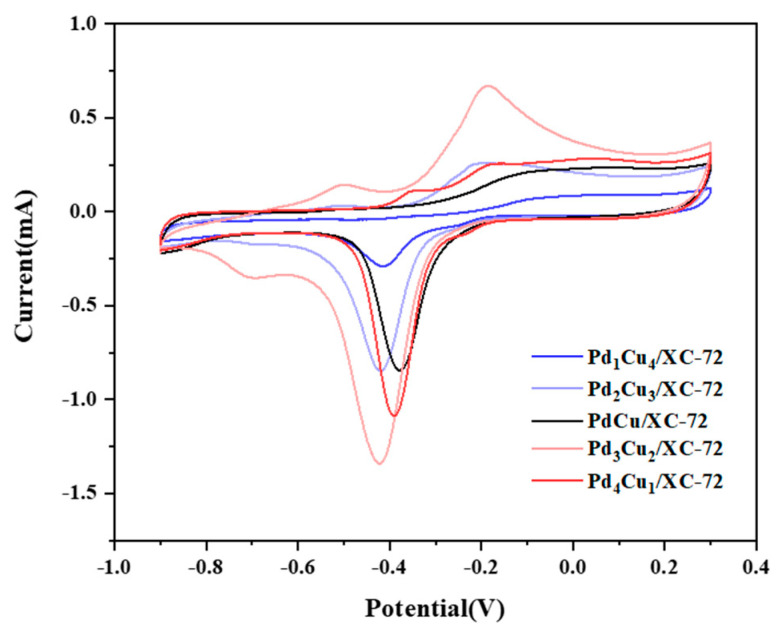
CVs in 1.0 M KOH + CH_3_OH-purged N_2_ at 50 mVs^−1^.

Figure 7 summarizes the electrochemical performance of five electrocatalysts. Cyclic voltammetry(CV) is performed on each of the Pd_1_Cu_4_/XC-72, Pd_2_Cu_3_/XC-72, PdCu/XC-72, Pd_3_Cu_2_/XC-72 and Pd_4_Cu_1_/XC-72 electrode in N_2_-saturated 1.0 M KOH. The PdO reduction peaks of the catalysts containing Cu occur at earlier potentials, as seen in Figure 6. This means less energy was needed to reduce PdO in the presence of Cu. This is probably the attraction of Pd to metal oxides like CuO, which provides more electronics to the Pd and PdO active sites. In other words, it is more difficult to reduce PdO oxides as CuO withdraws electrons from Pd.

The electrochemical active surface area (ECSA) is also an important index for evaluating the activity of electrocatalysts. In this work, the ESCA of different ratios of Pd_x_Cu_y_/XC-72 is estimated by integrating the Pd reduction peaks in order to find the Coulombic charge of each catalyst, and then following the proposed method of Rand and Wood (Equation (1))

ECSA = Q/SL(1)

where Q is the Coulombic charge (0.0536, 0.1316, 0.1173, 0.216562, and 0.1444 mC for Pd_1_Cu_4_/XC-72, Pd_2_Cu_3_/XC-72, PdCu/XC-72, Pd_3_Cu_2_/XC-72, and Pd_4_Cu_1_/XC-72, respectively), S is the Coulombic constant for a monolayer of Pd (0.424 mC cm^−2^), and L is the electrocatalyst loading (∼x μg (Pd)). The calculated ECSA Pd values decrease as follows: Pd_3_Cu_2_/XC-72 (2.384 m^2^ g_Pd_^−1^) > Pd_2_Cu_3_/XC-72 (1.961 m^2^ g_Pd_^−1^) > PdCu/XC-72 (1.47 m^2^ g_Pd_^−1^) > Pd_1_Cu_4_/XC-72 (1.428 m^2^ g_Pd_^−1^) > Pd_4_Cu_1_/XC-72 (1.305 m^2^ g_Pd_^−1^). These results reveal that Cu acts as a promoter to boost the electrochemical activity of Pd.

**Figure 8:**
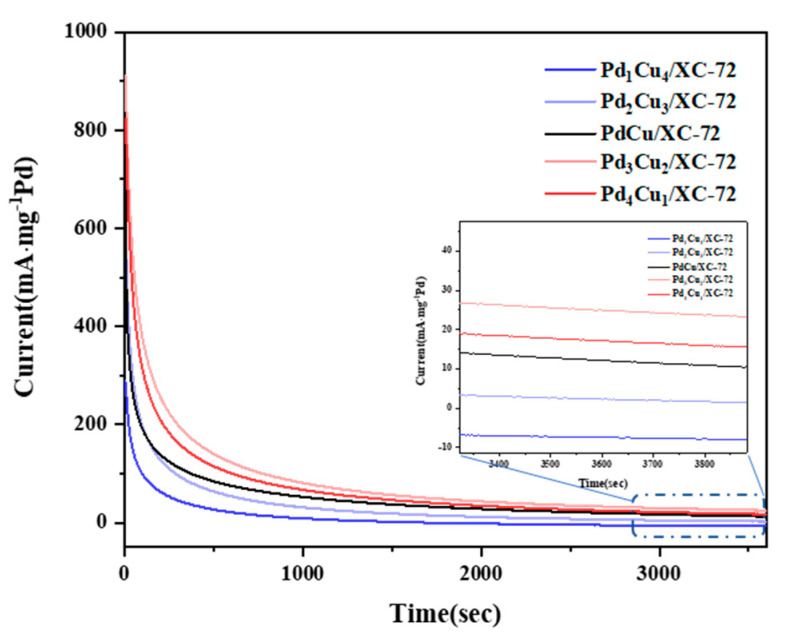
CAs in 1.0 M KOH + 1.0 M CH_3_OH−purged N_2_ at 50 mVs^−1^.

Chronoamperometry (CA) curves are recorded in 1.0 M CH_3_OH + 1.0 M KOH solution under a constant potential of −0.2 V, as shown in Figure 8. The high initial current density is caused by double-layer charging and abundant active sites for methanol activation. The current density dropped quickly within the first dozens of seconds due to the adsorption of CO produced from the MOR on the catalytic surface. 

After 500 s of testing, Pd_3_Cu_2_/XC-72 still had the highest ultimate current density (122.3 mA mg^−1^ Pd), further confirming that the Pd_3_Cu_2_/XC-72 nanocatalyst has the best electrocatalytic activity and long-term electrochemical durability for MOR. The synthesized Pd_3_Cu_2_/XC-72 nanoparticles have the advantages of large specific surface area, high alloying, strong electronic interactions between Pd and Cu atoms, a clear surface, and many active sites, which can significantly improve the electrocatalytic performance of MOR.

## 4. Conclusions

In summary, we studied a highly efficient palladium copper alloy catalyst with a coordination structure which was successfully synthesized by the liquid phase reduction method. During the synthesis, the ratios between Pd and Cu determined the alloying degree. The particular electronic characteristics endowed Pd_3_Cu_2_/XC-72 with large ECSA, fast mass transfer, and high self-stability, which contributed to the high electrocatalytic activity and excellent stability of Pd_3_Cu_2_/XC-72 for MOR in alkaline media. Additionally, Pd_3_Cu_2_/XC-72 exhibited enhanced MOR activity compared to Pd/C, indicating that the PdCu interface improved the MOR activity of Pd nanostructures. Experimental results demonstrated that the synergistic effect between Pd and Cu accelerated the oxidation of CO_ads_, which are also responsible for MOR activity/stability enhancement of Pd_3_Cu_2_/XC-72. This study provides new ideas for the design of highly active Pd electrocatalysts, and demonstrates the great potential of Pd_3_Cu_2_/XC-72 as a DMFCs anode electrocatalyst.

## Figures and Tables

**Table 1:** ICP-OES results for PdxCuy/XC-72 catalysts.

Sample	ICP Results (wt%)		Theory of Loads (wt%)		Theoretical Metal Molar Ratio (Pd: Cu)	Actual Metal Molar Ratio (Pd: Cu)
	Pd	Cu	Pd	Cu		
Pd_1_Cu_4_/XC-72	7.6	15.4	8.8	21.2	0.40	0.47
Pd_2_Cu_3_/XC-72	12.4	11.1	15.8	14.2	1.07	1.07
PdCu/XC-72	18.2	10.2	18.7	11.3	1.60	1.72
Pd_3_Cu_2_/XC-72	19.7	6.9	21.4	8.6	2.40	2.74
Pd_4_Cu_1_/XC-72	25.8	3.5	26.1	3.9	6.44	7.08

**Table 2:** Performance comparison of different palladium-based catalysts.

Catalyst	Test Condition	Scan Rate (mV/s)	Mass Activity (mA/mg)	Refs.
Pd_3_Cu_2_/XC-72	1 M KOH + 1 M Methanol	50 mV/s	1719 mA/mg	This Work
Pd/C	1 M KOH + 1 M methanol	50 mV/s	550 mA/mg	2019 [32]
Solid carbon sphere-supported Pd-CeO_2_ nanoparticles	1 M KOH + 1 M methanol	50 mV/s	900 mA/mg	2019 [32]
Pd_59_Cu_33_ Ru_8_ NSs	1 M KOH + 1 M methanol	50 mV/s	1660.8 mA/mg	2019 [33]
Pd-PdO PNTs-260	1 M KOH + 1 M methanol	50 mV/s	1111.3 mA/mg	2020 [34]
Pd_59_Fe_27_Pt_14_ NMs	1 M KOH + 1 M methanol	50 mV/s	1610 mA/mg	2020 [35]
Pd_3_Sn_2_ IMC	1 M KOH + 1 M methanol	50 mV/s	1300 mA/mg	2022 [36]
Pd/Se−Ti_3_C_2_	1 M KOH + 1 M methanol	50 mV/s	1046.2 mA/mg	2022 [27]
Pd_72_Cu_14_Co_14_/rGO	1 M KOH + 1 M methanol	50 mV/s	1062 mA/mg	2019 [31]

**Table 3:** Comparison of CO resistance of different catalysts.

	Pd_1_Cu_4_/XC-72	Pd_2_Cu_3_/XC-72	PdCu/XC-72	Pd_3_Cu_2_/XC-72	Pd_4_Cu_1_/XC-72
Mass activity	311 mA/mg_Pd_	1130 mA/mg_Pd_	1189 mA/mg_Pd_	1719 mA/mg_Pd_	1409 mA/mg_Pd_
*I_f_/I_b_*	3.41287	4.01877	1.999	5.307	4.785

## Data Availability

The data presented in this study are available upon request from the first author.
